# Supplementary figures and images for: Physiological impact of nanoporous acupuncture needles: Laser Doppler perfusion imaging in healthy volunteers
Source: PLoS One. 2019 Dec 11;14(12):e0226304. doi: 10.1371/journal.pone.0226304 (PMC6905535; doi:10.1371/journal.pone.0226304)

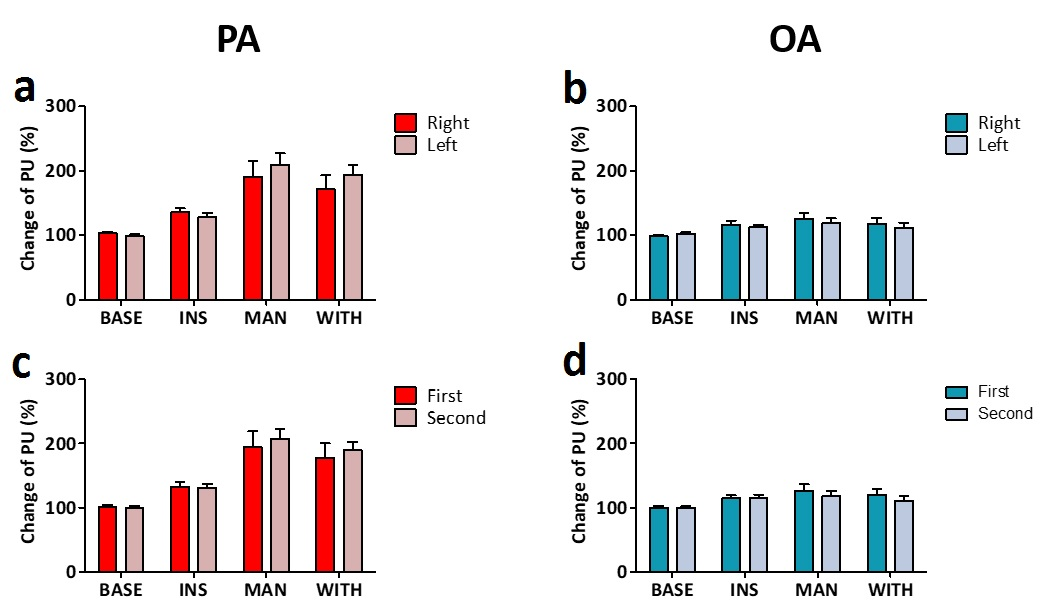

Supplement: S1 Fig — The changes of perfusion unit between the right and left legs from baseline for each session were compared by paired t-test. There were no significant differences between right and left blood perfusion in either the PA (a) or OA (b) group. The changes of perfusion unit by order from baseline for each session were compared by paired t-test. There were no significant differences in the PA (c) or OA (d) group. Values presented as means ± standard deviations. OA, ordinary acupuncture; PA, porous acupuncture. (TIF) [file pone.0226304.s001.tif]
