# Supplementary material for: Physiological impact of nanoporous acupuncture needles: Laser Doppler perfusion imaging in healthy volunteers
Source: PLoS One. 2019 Dec 11;14(12):e0226304. doi: 10.1371/journal.pone.0226304 (PMC6905535; doi:10.1371/journal.pone.0226304)
Supplement: S1 Table — This table was offered to participants to choose one or more sensations they felt during the experiment by each phase. (DOCX) [file pone.0226304.s003.docx]

**S1 Table. The sensation of acupuncture**

| **The sensation of acupuncture on each session** | |
| --- | --- |
| **(Korean)** | **(English)** |
| *뻐근한*  (Bugeunhan) | Dull |
| *우리한*  (Woorihan) | Numb |
| *시원한*  (Siwonhan) | Refreshing or relieving |
| *퍼져나가는*  (Pujunaganun) | Spreading out |
| *따뜻한*  (Daduthan) | Warm |
| *장기가 움직이는 듯한*  (Janggiga Umjikyinunduthan) | Activated digestion with bowel moving |
| *뭉친 근육을 풀어주는 듯한*  (Mungchin Gunyukul Puluhjoonunduthan) | Relieving sensation of tense or tight muscles |
| *묵직한*  (Mookjikhan) | Heavy |
| *살살 만지는*  (Salsalmanjinun) | A gentle (soft) touch |
| *막힌 것이 풀리는 듯한*  (Makhingutyi Pulinunduthan) | Surging opening flow of stuffed or choked feeling |
| *혈액순환이 되는 듯한*  (Hyuaksunhwanyi Doenunduthan) | Activated blood circulation |
| *누르는*  (Nurunun) | Compressing or pressuring |
| *기타* | Others |

This table was offered to participants to choose one or more sensations they felt during the experiment by each phase.
