# Supplementary material for: Physiological impact of nanoporous acupuncture needles: Laser Doppler perfusion imaging in healthy volunteers
Source: PLoS One. 2019 Dec 11;14(12):e0226304. doi: 10.1371/journal.pone.0226304 (PMC6905535; doi:10.1371/journal.pone.0226304)
Supplement: S2 Table — The t-test was performed to compare the differences between the first and the second sessions. Data represent Mean ± standard deviation. Min, minutes. (DOCX) [file pone.0226304.s004.docx]

**S2 Table. The comparisons in the changes of mean skin blood perfusion between the first and the second sessions**

| **Time (min)** | **PA (First)** | **PA (Second)** | ***p*-value** | **OA (First)** | **OA (Second)** | ***p*-value** |
| --- | --- | --- | --- | --- | --- | --- |
| **0** | 100.0 ± 0.0 | 100.0 ± 0.0 | 1.0000 | 100.0 ± 0.0 | 100.0 ± 0.0 | 1.0000 |
| **1** | 99.9 ± 10.7 | 100.4 ± 9.0 | 0.9155 | 103.3 ± 10.0 | 97.9 ± 6.9 | 0.1697 |
| **2** | 98.6 ± 13.2 | 102.2 ± 7.0 | 0.4330 | 104.2 ± 11.6 | 96.6 ± 5.8 | 0.0754 |
| **3** | 97.0 ± 13.0 | 104.3 ± 10.4 | 0.1677 | 104.7 ± 14.7 | 97.8 ± 10.4 | 0.2323 |
| **4** | 100.2 ± 18.9 | 103.9 ± 12.1 | 0.6007 | 102.6 ± 11.1 | 95.0 ± 8.1 | 0.0886 |
| **5** | 108.2 ± 15.6 | 110.8 ± 15.8 | 0.7142 | 100.4 ± 13.9 | 105.1 ± 19.1 | 0.5220 |
| **6** | 129.7 ± 33.7 | 118.4 ± 21.5 | 0.3681 | 107.1 ± 14.4 | 122.0 ± 28.4 | 0.1413 |
| **7** | 134.5 ± 36.1 | 126.3 ± 23.9 | 0.5385 | 111.9 ± 16.0 | 127.9 ± 28.0 | 0.1188 |
| **8** | 140.9 ± 29.1 | 126.0 ± 22.1 | 0.1990 | 107.5 ± 17.1 | 122.4 ± 19.2 | 0.0753 |
| **9** | 146.2 ± 28.5 | 128.3 ± 20.0 | 0.1099 | 108.9 ± 16.7 | 123.6 ± 20.7 | 0.0890 |
| **10** | 142.2 ±21.1 | 126.1 ± 22.3 | 0.1064 | 110.1 ± 18.8 | 122.1 ± 16.2 | 0.1339 |
| **11** | 140.1 ± 25.4 | 131.1 ± 19.5 | 0.3743 | 114.2 ± 19.1 | 115.5 ± 15.1 | 0.8598 |
| **12** | 143.5 ± 33.0 | 137.1 ± 26.6 | 0.6313 | 115.7 ± 17.5 | 118.4 ± 16.9 | 0.7210 |
| **13** | 142.0 ± 35.2 | 133.9 ± 27.1 | 0.5562 | 115.5 ± 22.7 | 119.1 ± 16.6 | 0.6909 |
| **14** | 143.1 ± 35.2 | 127.9 ± 24.3 | 0.2598 | 113.3 ± 22.3 | 114.8 ± 14.8 | 0.8590 |
| **15** | 187.9 ± 58.9 | 149.6 ± 43.0 | 0.1035 | 119.8 ± 29.9 | 129.3 ± 23.4 | 0.4301 |
| **16** | 204.7 ± 61.6 | 188.7 ± 66.3 | 0.5753 | 119.3 ± 28.8 | 129.0 ± 27.1 | 0.4367 |
| **17** | 217.2 ± 63.8 | 200.6 ± 80.1 | 0.6071 | 123.5 ± 31.2 | 130.5 ± 23.9 | 0.5768 |
| **18** | 220.9 ± 72.5 | 196.9 ± 77.0 | 0.4719 | 124.3 ± 32.8 | 129.0 ± 26.6 | 0.7254 |
| **19** | 218.7 ± 65.7 | 201.3 ± 84.4 | 0.6067 | 121.1 ± 27.7 | 129.8 ± 24.1 | 0.4523 |
| **20** | 210.8 ± 63.9 | 195.4 ± 81.8 | 0.6402 | 121.0 ± 38.7 | 122.7 ± 22.8 | 0.9074 |
| **21** | 210.5 ± 62.4 | 199.9 ± 88.5 | 0.7563 | 116.1 ± 32.0 | 124.9 ± 23.5 | 0.4887 |
| **22** | 202.6 ± 68.5 | 199.4 ± 83.6 | 0.9254 | 119.6 ± 36.9 | 117.0 ± 19.7 | 0.8442 |
| **23** | 201.1 ± 66.3 | 208.1 ± 88.8 | 0.8397 | 117.8 ± 36.4 | 119.0 ± 22.7 | 0.9328 |
| **24** | 204.2 ± 69.9 | 194.9 ± 90.9 | 0.7980 | 115.2 ± 40.3 | 118.5 ± 23.5 | 0.8237 |
| **25** | 193.8 ± 54.2 | 189.8 ± 70.4 | 0.8880 | 114.2 ± 37.3 | 118.3 ± 23.3 | 0.7702 |
| **26** | 192.3 ± 50.7 | 189.9 ± 65.3 | 0.9247 | 116.0 ± 33/4 | 114.3 ± 23.9 | 0.8932 |
| **27** | 187.3 ± 52.4 | 177.3 ± 63.0 | 0.6974 | 110.5 ± 22.8 | 117.1 ± 23.6 | 0.5267 |
| **28** | 177.7 ± 49.5 | 184.4 ± 78.2 | 0.8192 | 117.0 ± 30.0 | 113.5 ± 24.9 | 0.7769 |
| **29** | 170.9 ± 50.2 | 181.8 ± 73.7 | 0.6985 | 116.5 ± 32.5 | 116.2 ± 27.1 | 0.9828 |
| **30** | 172.6 ± 51.6 | 184.4 ± 70.6 | 0.6705 | 115.2 ± 29.6 | 116.7 ± 20.6 | 0.8953 |

The t-test was performed to compare the differences between the first and the second sessions. Data represent Mean ± standard deviation. Min, minutes.
