# Supplementary material for: Physiological impact of nanoporous acupuncture needles: Laser Doppler perfusion imaging in healthy volunteers
Source: PLoS One. 2019 Dec 11;14(12):e0226304. doi: 10.1371/journal.pone.0226304 (PMC6905535; doi:10.1371/journal.pone.0226304)
Supplement: Attachment — Submitted filename: Response to Reviewers.docx [file pone.0226304.s005.docx]

*Hi-Joon Park, K.M.D., Ph.D.*

*Studies of Translational Acupuncture Research*

*Acupuncture and Meridian Science Research Center, Kyung Hee University*

*26 Kyungheedae-ro, Dongdaemun-gu, Seoul 02447, Republic of Korea*

*Email:* [*acufind@khu.ac.kr*](mailto:acufind@khu.ac.kr)

*Tel: +82-2-961-0371; Fax: +82-2-961-9160*

October 29^th^, 2019

Re: Manuscript ID PONE-D-19-18282 Physiological impact of nanoporous acupuncture needles: laser Doppler perfusion imaging in healthy volunteers

Dear reviewers,

My colleagues and I greatly appreciate the comments and critiques of the reviewers and have incorporated their suggestions and recommendations into the manuscript. We wish to acknowledge the reviewers contributions.

- Reviewer #1

I thought the authors present an excellent piece of research on nanoporous acupuncture needles - there's definitely novelty and implications for use in clinical practice. However, I reckon this manuscript needs minor to major revisions with regard to (I) English language for clarity and style consistent with the standards of scientific / academic rigour (II) inadequate reporting of data from which inferences were drawn.

I have tried to highlight some of the points.

1. Lines 36-37 & 108-109: "In a randomized, controlled crossover trial" - is this a randomised crossover laboratory study?

As recommended, we added “clinical” between “crossover” and “trial” to “In a randomized, controlled crossover clinical trial” for clarity.

2. Line 43: "consequently" - or subsequently?

We replaced “consequently” with “subsequently”.

3. Lines 91-95: consider rephrasing the sentence - it appears too long and difficult to comprehend.

As recommended, we rephrased the sentence as following:

“Accordingly, porous acupuncture (PA), which is anodized to increase its surface area to a value approximately 20 times greater than conventional acupuncture needles, was developed to increase the physical friction applied to the surrounding tissue for higher stimulation intensity [36]. PA presented significantly improved therapeutic effect with more comfort in alcohol-dependent [36] and colorectal cancer animal models [37].

1. Line 101: "led to the enhanced therapeutic effect of acupuncture" - therapeutic effect appears vague, is it increased flow of blood in the microcirculation, pain-relief? consider for clarity?

As recommended, we added the phrase of “e.g. analgesic effect of acupuncture” for clarity.

5. Line 117: "Participants" - I think they are still volunteers at this stage. Once they enroll after signing consent, they become participants.

We replaced “Participants” with “Volunteers”.

6. Lines 117-121: I was wondering what is the reason for your eligibility criteria? Some references might help

We referred to the previous studies on the changes of local blood flow in response to acupuncture stimulation, in which volunteers with any history of cardiac, respiratory, neurologic, or hormonal diseases were excluded due to their possible influences on the study results. We added a systematic review [reference #39] including those previous studies as a reference to the “Participants” part under Methods Section.

7. Lines 122-125: Consider rephrasing the sentence – unclear

As recommended, we rephrased the sentence by omitting some unnecessary phrases as following:

“Detailed explanations of the experimental procedures that the recruits would be subjected to were given and the recruits provided informed consent for participation”.

8. Line 129: consider deleting "trial"

We deleted “trial”.

9. Line 133 & 152-156: "received PA on the left leg and, after 30 minutes of rest, received OA on the right leg" - I think I get the sense of it but some readers might find it difficult to follow - for e.g., was Doppler imaging done during these 30 mins? I think it would be easier to follow if there's a graph showing sequence of events

- We agree with the reviewer. As “30 mins” is a separation between the two experiment sessions, we added the related sentence in the “Experimental Design and Procedures” part under Methods Section as following:

“Participants were divided into four groups in random order and session orders were determined with a random number table generated by Excel (Microsoft, Redmond, WA, USA). Each experiment was separated by 30 minutes. Among the four groups, one group received PA on the left leg and, after 30 minutes of rest, received OA on the right leg, while another group first received PA on the right and then OA on the left. In the same manner, the other two groups respectively received either OA on the left followed by PA on the right or OA on the right followed by PA on the left (Fig 1a)”.

- In addition, we modified the graph showing sequence of events that readers could understand it easier by adding the phrase of “30 min rest” between each experiment (Fig 1a).

10. Lines 141-145: "history of alcohol ingestion, smoking, and medication" - I appreciate the information collected, think these could potentially have affected blood microcirculation? If these were not controlled, I'd consider making a note of it in the limitations section.

We agree with the reviewer that those could potentially affect blood microcirculation and therefore, participants were asked to refrain from consuming those things for 12 hours before the experiment. We added the related sentence to the “Experimental Design and Procedures” part under Methods Section as following:

“All participants refrained from consuming alcohol, caffeine, or medication for 12 hours before the experiment”.

11. Line 155: "consequently" or subsequently?

We replaced “consequently” with “subsequently”.

12. Line 166: could cleaning with alcohol have altered blood microcirculation?

Although it might have, we think there would be little impact on the study results because cleaning with alcohol was done prior to baseline and then, insertion of acupuncture was done after 5 mins of baseline measurement. Moreover, cleaning with alcohol was done equally to each session for all the participants.

13. Lines commencing 359: "study`" single inverted comma might have inadvertently been put?

It was an inadvertent mistake. We deleted the single inverted comma with some others which also have inadvertently been put.

Other points

14. Statistical analysis seem to have been performed appropriately however reporting data tables might be useful.

As recommended, for more clarity, we added a data table on the changes of mean skin blood perfusion for each session according to the order as S2 Table as following:

**S2 Table. The comparisons in the changes of mean skin blood perfusion between the first and the second sessions**

| **Time (min)** | **PA (First)** | **PA (Second)** | ***p*-value** | **OA (First)** | **OA (Second)** | ***p*-value** |
| --- | --- | --- | --- | --- | --- | --- |
| **0** | 100.0 ± 0.0 | 100.0 ± 0.0 | 1.0000 | 100.0 ± 0.0 | 100.0 ± 0.0 | 1.0000 |
| **1** | 99.9 ± 10.7 | 100.4 ± 9.0 | 0.9155 | 103.3 ± 10.0 | 97.9 ± 6.9 | 0.1697 |
| **2** | 98.6 ± 13.2 | 102.2 ± 7.0 | 0.4330 | 104.2 ± 11.6 | 96.6 ± 5.8 | 0.0754 |
| **3** | 97.0 ± 13.0 | 104.3 ± 10.4 | 0.1677 | 104.7 ± 14.7 | 97.8 ± 10.4 | 0.2323 |
| **4** | 100.2 ± 18.9 | 103.9 ± 12.1 | 0.6007 | 102.6 ± 11.1 | 95.0 ± 8.1 | 0.0886 |
| **5** | 108.2 ± 15.6 | 110.8 ± 15.8 | 0.7142 | 100.4 ± 13.9 | 105.1 ± 19.1 | 0.5220 |
| **6** | 129.7 ± 33.7 | 118.4 ± 21.5 | 0.3681 | 107.1 ± 14.4 | 122.0 ± 28.4 | 0.1413 |
| **7** | 134.5 ± 36.1 | 126.3 ± 23.9 | 0.5385 | 111.9 ± 16.0 | 127.9 ± 28.0 | 0.1188 |
| **8** | 140.9 ± 29.1 | 126.0 ± 22.1 | 0.1990 | 107.5 ± 17.1 | 122.4 ± 19.2 | 0.0753 |
| **9** | 146.2 ± 28.5 | 128.3 ± 20.0 | 0.1099 | 108.9 ± 16.7 | 123.6 ± 20.7 | 0.0890 |
| **10** | 142.2 ±21.1 | 126.1 ± 22.3 | 0.1064 | 110.1 ± 18.8 | 122.1 ± 16.2 | 0.1339 |
| **11** | 140.1 ± 25.4 | 131.1 ± 19.5 | 0.3743 | 114.2 ± 19.1 | 115.5 ± 15.1 | 0.8598 |
| **12** | 143.5 ± 33.0 | 137.1 ± 26.6 | 0.6313 | 115.7 ± 17.5 | 118.4 ± 16.9 | 0.7210 |
| **13** | 142.0 ± 35.2 | 133.9 ± 27.1 | 0.5562 | 115.5 ± 22.7 | 119.1 ± 16.6 | 0.6909 |
| **14** | 143.1 ± 35.2 | 127.9 ± 24.3 | 0.2598 | 113.3 ± 22.3 | 114.8 ± 14.8 | 0.8590 |
| **15** | 187.9 ± 58.9 | 149.6 ± 43.0 | 0.1035 | 119.8 ± 29.9 | 129.3 ± 23.4 | 0.4301 |
| **16** | 204.7 ± 61.6 | 188.7 ± 66.3 | 0.5753 | 119.3 ± 28.8 | 129.0 ± 27.1 | 0.4367 |
| **17** | 217.2 ± 63.8 | 200.6 ± 80.1 | 0.6071 | 123.5 ± 31.2 | 130.5 ± 23.9 | 0.5768 |
| **18** | 220.9 ± 72.5 | 196.9 ± 77.0 | 0.4719 | 124.3 ± 32.8 | 129.0 ± 26.6 | 0.7254 |
| **19** | 218.7 ± 65.7 | 201.3 ± 84.4 | 0.6067 | 121.1 ± 27.7 | 129.8 ± 24.1 | 0.4523 |
| **20** | 210.8 ± 63.9 | 195.4 ± 81.8 | 0.6402 | 121.0 ± 38.7 | 122.7 ± 22.8 | 0.9074 |
| **21** | 210.5 ± 62.4 | 199.9 ± 88.5 | 0.7563 | 116.1 ± 32.0 | 124.9 ± 23.5 | 0.4887 |
| **22** | 202.6 ± 68.5 | 199.4 ± 83.6 | 0.9254 | 119.6 ± 36.9 | 117.0 ± 19.7 | 0.8442 |
| **23** | 201.1 ± 66.3 | 208.1 ± 88.8 | 0.8397 | 117.8 ± 36.4 | 119.0 ± 22.7 | 0.9328 |
| **24** | 204.2 ± 69.9 | 194.9 ± 90.9 | 0.7980 | 115.2 ± 40.3 | 118.5 ± 23.5 | 0.8237 |
| **25** | 193.8 ± 54.2 | 189.8 ± 70.4 | 0.8880 | 114.2 ± 37.3 | 118.3 ± 23.3 | 0.7702 |
| **26** | 192.3 ± 50.7 | 189.9 ± 65.3 | 0.9247 | 116.0 ± 33/4 | 114.3 ± 23.9 | 0.8932 |
| **27** | 187.3 ± 52.4 | 177.3 ± 63.0 | 0.6974 | 110.5 ± 22.8 | 117.1 ± 23.6 | 0.5267 |
| **28** | 177.7 ± 49.5 | 184.4 ± 78.2 | 0.8192 | 117.0 ± 30.0 | 113.5 ± 24.9 | 0.7769 |
| **29** | 170.9 ± 50.2 | 181.8 ± 73.7 | 0.6985 | 116.5 ± 32.5 | 116.2 ± 27.1 | 0.9828 |
| **30** | 172.6 ± 51.6 | 184.4 ± 70.6 | 0.6705 | 115.2 ± 29.6 | 116.7 ± 20.6 | 0.8953 |

The t-test was performed to compare the differences between the first and the second sessions. Data represent Mean ± standard deviation. Min, minutes.

15. Other information missing such as was constraint randomisation used? What are the p values in Tables 2 & 3

- Randomization procedures are reported in the “Experimental Design and Procedures” part under Methods Section as following:

“This study’s design was a randomized crossover in which each participant served as his or her own control. Participants were divided into four groups in random order and session orders were determined with a random number table generated by Excel (Microsoft, Redmond, WA, USA)”.

- *P*-values < 0.05 were considered to be statistically significant and we added the *p* values in Tables 2 & 3 (*p* = 0.3239, 0.2904 each).

16. Although the authors have provided conclusion in the abstract, I think it is missing from the main text.

We added conclusion under Conclusions Section as following:

“The study results suggest the possibility of PA in clinical use and its applicability in further investigations, as PA increased local blood flow without any associated discomfort or pain”.

Reviewer #2

This study is a physiological assessment of one new kind of acupuncture (nanoporous acupuncture) compared with traditional acupuncture. Although, the study is innovative, the design of the study is not rigorous.

1. Firstly, the choose of healthy people is not enough since most of the subjects who seek acupuncture treatment is not healthy.

- We absolutely agree with the reviewer that it is not enough only with healthy volunteers. However, as the reviewer mentioned beneath, the effectiveness of porous acupuncture (PA) is still totally unknown. Moreover, the physiological impact and applicability in clinical use of PA have not yet been confirmed in any clinical trial. This study is the first clinical study using PA and at this time, it simply aimed to explore its physiological impact, the possibility of PA in clinical use and its applicability in further investigations. Based on the results of this study, further clinical studies on the clinical efficacy and safety of PA with participants having any clinical symptom or disease, which certainly needed, could be performed.

- We mentioned this point as one of the limitations under Discussion Section as following:

“Finally, our study was not targeted to any clinical symptom or disease; we could only conclude that PA has a more significant physiological impact on local blood flow change than OA, which might be related to the higher stimulation intensity and enhanced performance. To establish the relation of the effect on the blood flow change with the stimulation intensity and the clinical performance, further study simultaneously investigating the clinical effects of PA is needed”.

- To clearly determine the aim of this study that it simply aimed to explore its physiological impact, the possibility of PA in clinical use and its applicability in further investigations rather than to investigate the clinical efficacy and safety, we added some related phrases in the “Background” parts under Abstract as following:

“In a randomized, controlled crossover clinical trial, we investigated the effects of PA on the change in local blood flow using laser Doppler perfusion imaging and considered the sensation of pain intensity and discomfort severity using a visual analogue scale (VAS) to explore its physiological impact and the possibility of PA in clinical use”.

2. Secondly, only one acupoint-ST36 for the assessment is not consistent with the real situation. Usually, we will use more than 10 acupoints per treatment and their blood flow may interact with each other.

- We agree with the reviewer that using just one acupoint is not consistent with the real situation. However, as we mentioned above, at this time, we simply aimed to explore its local physiological impact induced by insertion itself, with porous acupuncture (PA) needles. We referred to the previous studies on the changes of local blood flow in response to acupuncture stimulation [reference #39], of which most of the included studies used just one acupoint or local point as measurement site.

- Still, it is one of the limitations of this study, of which the results can only reflect local physiological impact on just one acupoint without any consideration of possible interactions with other acupoints. Further clinical studies considering the interactions between multiple acupoints would be certainly needed, based on the results of studies with one local acupoint. We added this point as one of the limitations of this study under Discussion Section as following:

“Moreover, studies using multiple acupoints of various anatomical locations considering possible interactions with each other would be certainly needed based on this study results with one local acupoint”.

3. Thirdly, the effectiveness of this kind of acupuncture is totally unknown and the underlying mechanism of acupuncture is complex and only test blood flow is too far from the traditional acupuncture.

We agree with the reviewer that we need to further investigate the underlying complex mechanism of acupuncture. As we mentioned above, based on this study results of local physiological impact and clinical applicability of porous acupuncture (PA), we’re planning to perform further study simultaneously investigating the clinical effects and safety of PA with participants having any clinical symptom or disease on multiple acupoints, which certainly needed, along with various physiological parameters. However, at this time, we think this study is essential because it aimed to explore the possibility of PA in clinical use and its applicability in further investigations. We added this point as one of the future studies needed under Discussion Section as following:

“In addition, research on the specific mechanisms designed to explain why PA had different physiological influences on local blood flow, e.g. vasoactive substances, are necessary…Studies investigating other physiological parameters may be meaningful as future endeavors”.

Sincerely yours,

Hi-Joon Park, KMD, Ph. D.
